# Risk factors for the development of triple-negative breast cancer versus non-triple-negative breast cancer: a case–control study

**DOI:** 10.1038/s41598-023-40443-8

**Published:** 2023-08-20

**Authors:** Shona Nag, Rajesh Dikshit, Sangeeta Desai, Anupama Mane, Sharayu Mhatre, Rakesh Neve, Mamta Gurav, Neelambari Bhosale, Prema Perumal, Yogesh Kembhavi, Dinesh Jethwa, Rajendra Badwe, Sudeep Gupta

**Affiliations:** 1 Medical Oncology, Jehangir Clinical Development Center, Pune, India; 2 Tata Memorial Centre, Homi Bhabha National Institute, Mumbai, India; 3 Pathology, Tata Memorial Centre, Homi Bhabha National Institute, Mumbai, India; 4 Surgical Oncology, Jehangir Clinical Development Center, Pune, India; 5 Medical Oncology, Tata Memorial Centre, Homi Bhabha National Institute, Mumbai, India; 6 Surgical Oncology, Tata Memorial Centre, Homi Bhabha National Institute, Mumbai, India; 7 Tata Memorial Hospital/Centre, Room 1109, Homi Bhabha Block, Parel, Mumbai, 400012 India

**Keywords:** Risk factors, Cancer, Breast cancer, Cancer epidemiology

## Abstract

The risk factors for breast cancer have been defined in several studies but there is deficient data for specific subtypes. We report here the pathological characteristics of a breast cancer cohort and risk factors for patients with triple-negative disease. In this case–control study, a prospective breast cancer cohort was evaluated for demographic, reproductive, obesity-related and other risk factors using a validated questionnaire. Tumors were characterized for routine pathological characteristics and immunohistochemical markers of basal-like breast cancer. Patients with triple-negative breast cancer (TNBC) constituted cases and those with non-TNBC were controls. Odds ratios (OR) were calculated for each risk factor and independent associations were tested in an unconditional logistic regression analysis. Between 2011 and 2014, 1146 patients were recruited, of whom 912 [TNBC 266 (29.1%), non-TNBC 646 (70.9%)] with sufficient pathology material were analysed. Reproductive factors of parity, breastfeeding, age-at-menarche, age at first full-term pregnancy and oral contraceptive use were not significantly associated with TNBC. Higher body mass index (BMI > 24.9 vs ≤ 24.9, OR 0.89, 95%CI 0.63–1.24, p = 0.49) was not significantly associated while lesser waist circumference (> 80 cm vs ≤ 80 cm, OR 0.64, 95%CI 0.45–0.9, p = 0.012) and lower waist-to-hip ratio were significantly associated (> 0.85 vs ≤ 0.85, OR 0.72, 95%CI 0.51–1.0, p = 0.056), with TNBC. History of tobacco use was not significantly associated while lower socio-economic status was borderline associated with TNBC (socio-economic category > 5 versus ≤ 5, OR 0.73, 95%CI 0.50–1.06, p = 0.106). No factor was significant after adjustment for covariates. Central obesity seems to be preferentially associated with non-TNBC, and lower socio-economic status with TNBC in India, while most other conventional risk factors of breast cancer show no significant association with TNBC versus non-TNBC.

## Introduction

Breast cancer is the most common cancer among women in urban India, with its incidence recently surpassing that of cervical cancer^1,2^. Epidemiological studies have shown an association between breast cancer and smoking, alcohol consumption, a high-fat diet, reproductive factors, and socioeconomic status, which may explain its more frequent occurrence among women with a Western lifestyle. However, the risk factors for specific pathological and molecular subtypes of breast cancer have not been accurately defined. Therefore, the differential effect of risk factors on breast cancer subtypes, if any, remains unclear. A few studies have evaluated the risk factors for estrogen receptor negative breast cancer and suggested that higher parity and younger age at first child-birth may be associated with higher risk of developing this type^3^.

The proportion of estrogen receptor (ER)-positive breast cancer in Indian women appears to be lower (about 45–60%) than that in their European and American counterparts^4,5^. Accordingly, the fraction of patients with triple-negative breast cancer has been reported to be higher (25–30%) in patients from India and other developing countries^6,7^. The differences in hormone receptor positivity between Indian and Caucasian patients could be a real ethnic variation or it could be a result of lower average age at diagnosis.

Hence, we undertook this study to identify the risk factors for triple negative breast cancer using a case–control design in patients with carefully characterised pathological breast cancer subtypes. We also report the detailed pathological characterisation of breast tumours from the same cohort of patients.

## Methods

### Study design and patients

This was a prospective case–control study to elucidate the risk factors associated with the three major subtypes of breast cancer wherein patients with triple-negative breast cancer were considered to be cases while those with estrogen and/or progesterone receptor positive and HER2 negative and estrogen and progesterone receptor any status but HER2 positive or amplified, respectively, was considered as a common control group.

Enrolment in the study was determined at the first presentation of patients to either institution. Patients eligible for the study were women between 18 and 70 years of age diagnosed with invasive breast cancer who had to be treatment naïve except for surgery for the primary tumour. Those who had received neoadjuvant or adjuvant systemic therapy of any type were excluded, as also those with treatment for metastatic disease. If patients presented before surgery, core biopsy tissue was required for immunohistochemistry and molecular studies. If operated, the availability of paraffin-embedded blocks of the surgical specimen was essential for further studies. Patients should have been willing to provide informed consent for inclusion into the study including consent for blood samples for EBV and HPV testing.

The study was designed by faculty members of the breast cancer groups of both institutions and approved by the Institutional Ethics Committee of Tata Memorial Centre and the Ethics Committee of Jehangir Clinical Development Center. All participants provided written informed consent before study participation. All research procedures were performed in accordance with the Declaration of Helsinki and local regulations.

### Procedures

A detailed questionnaire that had previously been validated was administered to all participants at the time of study inclusion. Data on age, menopausal status, residential address (urban versus rural), and contact details were collected. Information was also obtained on the following potential risk factors for breast cancer: socioeconomic status, tobacco use, alcohol consumption, diet (predominantly vegetarian vs non-vegetarian), number of pregnancies up to or beyond the stage of viability (28 weeks), age at first childbirth, age at menarche/menopause, history of breastfeeding, family history of breast or ovarian cancers, and number of members living in the same household for the preceding life period. The data for residence included any place of residence lived in for a minimum period of 1 year. The detailed definition of these risk factors and the methodology of their collection followed the methods described in previous epidemiologic studies and are described in the study protocol and questionnaire Specifically, some risk factors were further dichotomised using acceptable cut-offs as described in previous epidemiologic literature^8^.

The following clinical and pathological parameters were recorded: weight, height clinical and (if available) pathological tumour size, clinical and (if available) pathological node status, grade, presence of lymphovascular invasion, immunohistochemistry (IHC)-based estrogen receptor (ER), progesterone receptor (PR), human epidermal growth factor receptor 2 (HER2) status, and Ki-67 index. Additionally, all triple-negative tumours were analysed for expression of core basal markers of cytokeratins (CK) 5/6, CK14, CK17, and epidermal growth factor receptor (EGFR) using standard immunohistochemical technique. Fluorescence in situ hybridisation (FISH) was performed if the HER2 score was 2+ on IHC. All pathological evaluations were performed under the supervision of a single experienced breast pathologist at the Tata Memorial Centre, Mumbai. The standard recommendations for staging and diagnosis were followed in the patient work-up before therapy initiation.

### Statistical analysis

The association of various risk factors with the subtypes was calculated using odds ratios (ORs) for exposure to each risk factor for triple-negative breast cancer (TNBC) cases vs non-TNBC controls. The individual OR was obtained using an unconditional logistic regression model. We estimated a sample size of 1000 to have 80% power to detect a minimum OR of 1.65 assuming 1 − ά = 95% and the prevalence of exposure to be 15%. The study was well-powered to detect an OR of 2.0 for exposure even with a very low prevalence among controls (prevalence = 10%, 1 − ά = 95%, and 1 − β = 80%). In a sample of 1000, the estimated proportions of patients with triple-negative, hormone receptor-positive and HER2 negative, and HER2-positive breast cancer were assumed to be 20–30%, 50%, and 20–30%, respectively.

Statistical analysis was performed using STATA version 15.0. All the variables of interest were cross-tabulated with the case–control status of the patients. OR and the corresponding 95% confidence intervals (CIs) for each risk factor under consideration were estimated using unconditional logistic regression models. Odds ratios were tabulated without any adjustment and after adjustment for covariates.

## Results

### Clinicopathological characteristics

**Figure 1:**
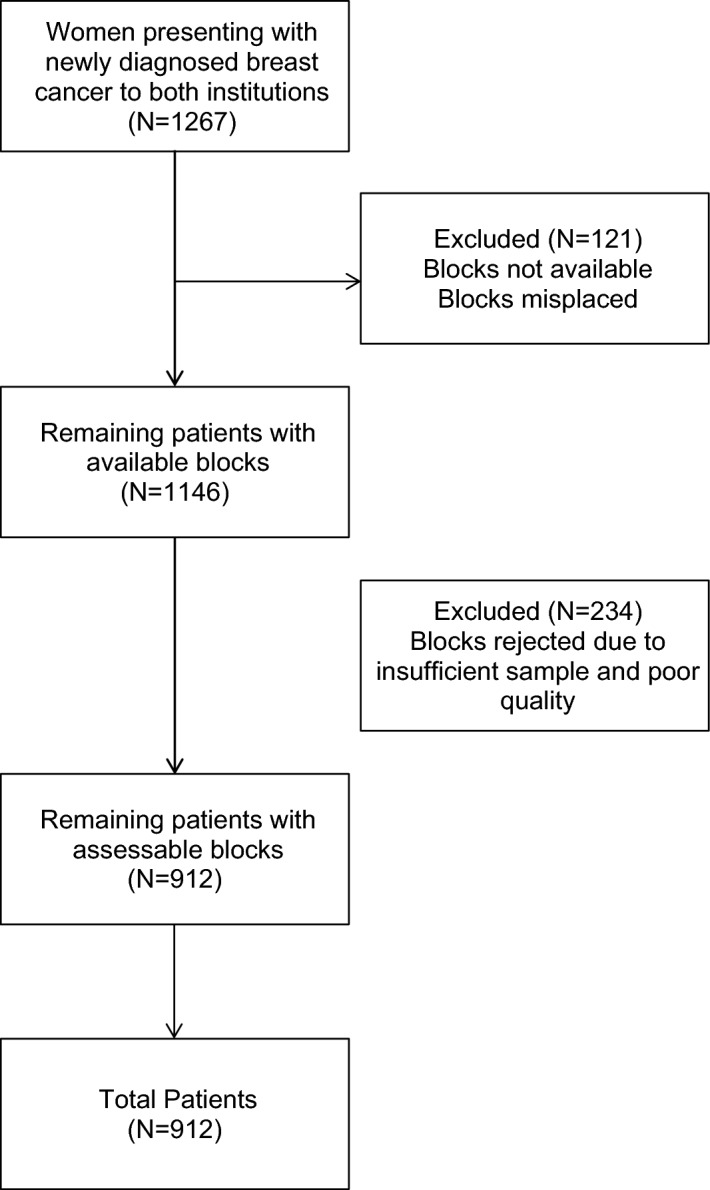
Enrolment.

This was a case–control study performed among newly diagnosed, previously untreated patients with invasive breast cancer of any stage who presented to two urban hospitals in Mumbai and Pune between July 2011 and December 2014 (n = 1267). Tissue samples for 355 patients were inadequate because the samples had been processed outside the two institutions and were either insufficient in quantity or had poor quality. Tissue samples for the remaining 912 patients were suitable for immunohistochemistry (IHC), and these patients were included in the study (Fig. 1). Patients with triple-negative breast cancer were regarded as the case group, while patients with hormone receptor-positive and HER2-negative breast cancer and those with any hormone receptor status and HER2-positive breast cancer served as the control group.

The clinical and pathological tumour data for the 912 patients included in the study are presented in Tables 1 and 2.

**Table 1:** Distribution of clinicopathological features and immunohistochemistry-based breast cancer subtypes (n = 912).

Median age, years, median (range)	47 (23–85)	
Clinicopathological features	Number	Percentage (%)
Tumor size		
T0	19	2.0
T1	143	15.6
T2	483	52.9
T3	126	13.8
T4	86	9.4
Tx	55	6.0
Nodal status		
N0	283	28.8
N1	299	32.7
N2	176	19.2
N3	102	11.1
Nx	52	5.7
Grade		
GI	15	1.6
GII	181	19.8
GIII	715	78.3
Gx	1	
Metastasis		
Yes	170	18.6
No	734	80.4
Unknown	8	0.8
Immunohistochemistry and subtype distribution		
ER		
Positive	494	54.0
Negative	418	46.0
ER positive subtype (N = 392)		
ER+/PR+	308	78.5
ER+/PR−	77	19.6
ER−/PR+	7	1.7
HER2 positive subtypes (N = 254)^a^		
ER+	109	42.9
ER−	145	57.0
Triple negative subtype (N = 266)		
ER/PR/HER2-	266	29.1

^a^4 FISH positive and HER2+ IHC.

ER: oestrogen receptor; PR: progesterone receptor; HER2: human epidermal growth factor receptor 2; FISH: fluorescence in situ hybridization.

**Table 2:** Distribution of core basal markers in patients with triple-negative breast cancer.

Core basal markers	Triple-negative tumors (N = 266)	Percentage (%)
CK5/6	172	64.6
CK14	117	43.9
CK17	138	51.8
CK 5/6 and CK14	106	39.8
CK 5/6 and CK17	122	45.8
CK 5/6, EGFR, CK14 and/or CK17	200	75.2

CK: cytokeratin.

The median age of these patients was 47 years (range, 23 to 85 years), 112 (23.2%) patients had T3 or T4 disease at diagnosis, 577 (63.2%) had pathologically confirmed axillary lymph node positive disease, 494 patients had ER-positive disease (54.0%) and 418 were ER-negative (46.0%). Of the hormone receptor-positive cases, 308 (78.5%) patients were both ER and PR positive and HER2 negative, 77 (19.6%) had ER positive and PR negative disease, and 7 (1.7%) had ER negative and PR positive tumors. The number of patients with HER2-positive disease (IHC 3+ or FISH amplified) was 254 (27.9%), of whom 109 (42.9%) were ER-positive and 145 (57%) were ER-negative. TNBC was present in 266 (29.1%) patients, of whom 106 (39.8%) expressed both CK5/6 and CK14, 122 (45.8%) expressed CK5/6 and CK17, while 200 (75.2%) expressed three or more core basal markers i.e. CK5/6, EGFR, CK14 and/or CK17.

### Association of risk factors with breast cancer subtypes

Of the 912 patients whose blocks were analysed by IHC, 905 completed the questionnaire satisfactorily. This included 651 patients with non-TNBC and 254 with TNBC. Table 3 shows the odds ratios of various risk factors for TNBC versus non-TNBC controls. In univariable analysis, no reproductive factor was significantly associated with TNBC versus non-TNBC, including number of full-term pregnancies (> 3 versus ≤ 3 pregnancies, OR 1.35, 95% CI 0.89–2.05, p = 0.149), breastfeeding (ever versus never, OR 1.16, 95% CI 0.48–2.80, p = 0.73), age at menarche (> 13 years versus ≤ 13 years, OR 1.33, 95% CI 0.95–1.87, p = 0.088), age at first full-term pregnancy (> 24 years versus ≤ 24 years, OR 0.95, 95% CI 0.67–1.35), and oral contraceptive use (ever vs never use, OR 0.80, 95% CI 0.49–1.59, p = 0.7). Among body size-related factors, higher body mass index (BMI > 24.9 vs ≤ 24.9, OR 0.89, 95% CI 0.63–1.24, p = 0.49) was not significantly associated, while lesser waist circumference (> 80 cm vs ≤ 80 cm, OR 0.64, 95% CI 0.45–0.9, p = 0.012) and lower waist-to-hip ratio (> 0.85 vs ≤ 0.85, OR 0.72, 95% CI 0.51–1.0, p = 0.056) were significantly associated with TNBC versus non-TNBC cancers. History of tobacco chewing or smoking (never use vs ever use, OR 1.3, 95%CI 0.35–2.00, p = 0.219) was not significantly associated with TNBC versus non-TNBC cancers. Lower socio-economic status was borderline associated with TNBC (socio-economic category > 5 versus ≤ 5, OR 0.73, 95%CI 0.50–1.06, p = 0.106). In the multivariable logistic regression analysis, no factor was significantly associated with TNBC versus non-TNBC (Table 3).

**Table 3:** Odds ratio for risk factors of triple-negative breast cancers compared with non-triple negative controls.

Variables	Categories	N (case/control)	OR^a^ (95%CI)	p-value	OR^b^ (95%CI)	p-value
Height^c^ (in cm)	< 150	93/224	1.00		1.00	
	≥ 150	153/391	0.88 (0.63–1.23)	0.466	1.03 (0.71–1.49)	0.875
Body mass index (in kg/m^2^)	≤ 24.9	120/275	1.00		1.00	
	> 24.9	134/376	0.89 (0.63–1.24)	0.496	0.94 (0.65–1.34)	0.750
Waist circumference^d^ (in cm)	≤ 80	91/177	1.00		1.00	
	> 80	157/434	0.64 (0.45–0.90)	0.012	0.63 (0.40–1.00)	0.053
Waist to hip ratio^d^	≤ 0.85	119/255	1.00		1.00	
	> 0.85	129/353	0.72 (0.51–1.00)	0.056	0.76 (0.53–1.08)	0.135
Age at menarche (in years)	≤ 13	93/274	1.00		1.00	
	> 13	156/357	1.33 (0.95–1.87)	0.088	1.31 (0.91–1.88)	0.144
Number of full-term pregnancies^e^	≤ 3	184/494	1.00		1.00	
	> 3	58/121	1.35 (0.89–2.05)	0.149	1.20 (0.75–1.91)	0.442
Breast feeding	Ever	7/25	1.00		1.00	
	Never	231/586	1.16 (0.48–2.80)	0.733	1.35 (0.51–3.61)	0.540
Age at first full-term pregnancy^f^ (in years)	≤ 24	149/367	1.00		1.00	
	> 24	88/244	0.95 (0.67–1.35)	0.809	1.02 (0.68–1.53)	0.894
Spontaneous abortion	No	201/515				
	Yes	42/104	1.11 (0.73–1.69)	0.619	1.23 (0.79–1.92)	0.346
Time between menarche and first full-term pregnancy^f^ (in years)	< 10	146/361	1.00		1.00	
	≥ 10	88/240	0.99 (0.69–1.40)	0.957	1.12 (0.75–1.67)	0.557
Induced abortion	No	179/439	1.00		1.00	
	Yes	21/66	0.82 (0.47–1.43)	0.500	0.95 (0.53–1.69)	0.863
Current residence	Rural	74/174	1.00		1.00	
	Urban	160/439	0.84 (0.58–1.20)	0.343	0.92 (0.62–1.37)	0.710
Tobacco use	Never	200/533	1.00		1.00	
	Ever	49/97	1.30 (0.85–2.00)	0.219	1.10 (0.69–1.74)	0.681
Oral contraceptives	Never	215/555	1.00		1.00	
	Ever	20/58	0.89 (0.49–1.59)	0.702	1.01 (0.54–1.89)	0.961
Family history of breast/ovarian cancer	No	241/619	1.00		1.00	
	Yes	10/23	1.19 (0.53–2.68)	0.633	1.49 (0.63–3.50)	0.352
Family history of any cancer	No	214/534	1.00		1.00	
	Yes	35/107	0.81 (0.51–1.27)	0.363	0.82 (0.50–1.34)	0.436
Socio-economic status (Modified Kuppuswamy’s Socioeconomic Scale)	≤ 5	68/152	1.00		1.00	
	> 5	182/484	0.73 (0.50–1.06)	0.106	0.76 (0.50–1.06)	0.213

OR: odds ratio; CI: confidence interval.

^a^Adjusted for age and region of residence.

^b^Adjusted for age, region of residence, age at first full-term pregnancy, education, waist-to-hip ratio, height, menopausal status, current rural–urban status, and total number of induced and spontaneous abortions.

^c^Adjusted for weight instead of height.

^d^Adjusted for body mass index instead of height and waist-to-hip ratio.

^e^Not adjusted for age at first full-term pregnancy.

^f^Adjusted for number of pregnancies instead of age at first full-term pregnancy.

## Discussion

Our results in a breast cancer patient cohort from two tertiary care cancer centres in urban India suggest that triple negative breast cancer constitutes a higher proportion of cases compared with that reported from developed countries and that TNBC phenotype is not significantly differentially associated with reproductive or body size related risk factors, compared with non-TNBC phenotype. This is one of the few studies that has prospectively analysed the association of breast cancer receptor-based subtypes with risk factors in a case-case analysis.

It is worth noting that our study was designed to evaluate the association of risk factors using TNBC patients as cases and non-TNBC patients as controls, which was meant to bring out differential predispositions, if any, to these subtypes of breast cancer in the Indian population. This also means that our results cannot be directly compared with other studies that tested the associations between patients with specific breast cancer subtypes using women without breast cancer as controls. Importantly, our results imply that risk factor modification strategies do not need to be specifically tailored for breast cancer subtypes and that a broad strategy is likely to be effective in modifying the population-level predisposition to all types of breast cancer, with the possible exception of parity, as discussed below.

One important previous study has suggested that high parity could be a risk factor for triple negative breast cancer^9^, although it is traditionally considered a protective factor for breast cancer. Our results suggest that parity is not significantly differentially associated with triple negative breast cancer compared with non-TNBC although the OR was 1.35. Given the limited power of finding risk factor associations in a case-case analysis, high parity being associated with TNBC remains a possibility, based on our results. There was no significant differential association of other reproductive risk factors like age at first full term pregnancy, age at menarche and breast feeding with TNBC, suggesting that these factors are likely to be similarly operative in predisposition to all types of breast cancer.

We did not find BMI to be differentially associated with TNBC compared with non-TNBC while a lower waist-to-hip ratio was borderline significantly associated with TNBC compared with non-TNBC. Some studies have suggested that a higher waist-to-hip ratio is associated with the risk of hormone receptor-positive breast cancer. In our study, we have reported that a lower waist-to-hip ratio was associated with the risk of TNBC. This is because ours was a case–control study wherein TNBC patients were cases and non-TNBC patients were controls. Therefore, all risk factor associations are preferential associations with TNBC compared with the non-TNBC subtype. An association of lower waist-to-hip ratio with TNBC in our study is consistent with literature reports of higher waist-to-hip ratio being associated with estrogen receptor-positive breast cancer. However, this would still be consistent with an overall association of a higher waist-to-hip ratio with the risk of TNBC when healthy individuals are used as controls, albeit to a lesser extent than estrogen receptor-positive disease. The Carolina study^10^ found an association of waist-hip ratio with TNBC among both premenopausal and post-menopausal women. It is likely that central obesity as measured by waist-to-hip ratio is a more accurate descriptor of the underlying metabolic predisposition to breast cancer compared with BMI because it considers not only the total body fat composition but also its distribution^11^. Other studies have variably found an association of TNBC with various measures of body weight^12–17^. Since our study used non-TNBC patients as control, the association of waist circumference and waist-to-hip ratio in our results suggests that central obesity is preferentially associated with non-TNBC, especially hormone receptor-positive disease, which constituted a high proportion of our non-TNBC controls in our study.

We also collected data on tobacco chewing, a somewhat unique form of tobacco used in India. It was not significantly associated with breast cancer subtypes. Interestingly, lower socioeconomic status was borderline associated with TNBC, the reasons for which are unclear but could reflect the impact of other factors. However, since this association was not statistically significant in univariable and multivariable analyses, it could result from chance.

Our study confirms previous reports that triple-negative breast cancer phenotype constitutes a higher proportion of patients^6^ in India. We also found that a high proportion of TNBC tumors express the immunohistochemical markers, i.e. CK5/6, CK14, CK17 or EGFR of basal-like cancers. A previous report in a subset of patients from this study reported a high prevalence of Epstein–Barr Virus (EBV) in the tumor cells of TNBC tumors^18^.

Our study has several strengths. It was a prospective study with a large sample size that included patients who presented to two large tertiary care hospitals. The tissue samples were processed by a central laboratory, and all the tumours were subtyped by a single experienced pathologist at a tertiary cancer centre.

Nevertheless, our study also had a few limitations. Because women with non-TNBC cancers were used as controls, the odds ratios in our study indicate the association of each risk factor with these phenotypes. The absolute risk association of each factor with TNBC can only be analysed in a study that includes healthy persons as the control population. Moreover, we did not perform germline sequencing for variants that predispose to breast cancer, like *BRCA1* and *BRCA2* pathogenic variants. This information might be useful in evaluating the interaction between germline predisposition and risk factors.

In conclusion, the results of our case–control analysis of the association of risk factors with breast cancer phenotypes suggests that lower waist-to-hip ratio, lower socio-economic status and possibly high parity could be differentially associated with triple-negative breast cancer compared with non-TNBC cancers, although these associations were not statistically significant. Most other reproductive and non-reproductive risk factors showed no significant association with breast cancer phenotypes. Broad risk factor modification strategies are likely to be useful as population-level interventions.

## Data Availability

The datasets generated and/or analysed during the current study are not publicly available due to restrictions on sharing data without IRB approval but are available from the corresponding author on reasonable request after due approval of the Ethics Committees of the participating institutions.
